# Early life adversity shapes neural circuit function during sensitive postnatal developmental periods

**DOI:** 10.1038/s41398-022-02092-9

**Published:** 2022-08-01

**Authors:** Lauren Malave, Milenna T. van Dijk, Christoph Anacker

**Affiliations:** 1 Division of Systems Neuroscience, Research Foundation for Mental Hygiene, Inc. (RFMH)/New York State Psychiatric Institute (NYSPI), Department of Psychiatry, Columbia University Irving Medical Center (CUIMC), New York, NY 10032 USA; 2 Division of Translational Epidemiology, Research Foundation for Mental Hygiene, Inc. (RFMH)/New York State Psychiatric Institute (NYSPI), Department of Psychiatry, Columbia University Irving Medical Center (CUIMC), New York, NY 10032 USA; 3 Sackler Institute for Developmental Psychobiology, Research Foundation for Mental Hygiene, Inc. (RFMH)/New York State Psychiatric Institute (NYSPI), Department of Psychiatry, Columbia University Irving Medical Center (CUIMC), New York, NY 10032 USA; 4 Columbia University Stem Cell Initiative (CSCI), Columbia University Irving Medical Center (CUIMC), New York, NY 10032 USA

**Keywords:** Depression, Epigenetics in the nervous system

## Abstract

Early life adversity (ELA) is a major risk factor for mental illness, but the neurobiological mechanisms by which ELA increases the risk for future psychopathology are still poorly understood. Brain development is particularly malleable during prenatal and early postnatal life, when complex neural circuits are being formed and refined through an interplay of excitatory and inhibitory neural input, synaptogenesis, synaptic pruning, myelination, and neurogenesis. Adversity that influences these processes during sensitive periods of development can thus have long-lasting and pervasive effects on neural circuit maturation. In this review, we will discuss clinical and preclinical evidence for the impact of ELA on neural circuit formation with a focus on the early postnatal period, and how long-lasting impairments in these circuits can affect future behavior. We provide converging evidence from human and animal studies on how ELA alters the functional development of brain regions, neural circuits, and neurotransmitter systems that are crucial for cognition and affective behavior, including the hippocampus, the hypothalamus-pituitary-adrenal (HPA) axis, neural networks of fear responses and cognition, and the serotonin (5-HT) system. We also discuss how gene-by-environment (GxE) interactions can determine individual differences in susceptibility and resilience to ELA, as well as molecular pathways by which ELA regulates neural circuit development, for which we emphasize epigenetic mechanisms. Understanding the molecular and neurobiological mechanisms underlying ELA effects on brain function and psychopathology during early postnatal sensitive periods may have great potential to advance strategies to better treat or prevent psychiatric disorders that have their origin early in life.

## Introduction

Early life adversity (ELA) is the exposure to negative experiences early in life, and includes adverse childhood experiences (ACEs) of different severity, such as war, natural disasters, physical or sexual abuse, malnourishment, parental psychopathology, and adverse parenting behaviors such as maltreatment, neglect, distant parent-child relationships, and unpredictable or disorganized parental care. According to epidemiological research, between one and two thirds of children will be exposed to at least one ACE [1, 2], and around 1 in 6 children will experience more severe exposure to four or more ACEs [3]. When experienced early in life, adversity can have particularly potent and long-lasting effects on the brain, in part because it affects neural development during sensitive periods when crucial neural connections are being formed. As a result, ELA increases risk for psychopathology in childhood and in adulthood, including cognitive impairments, decreased resilience to future stressors, conduct disorder, substance use disorder, depression and anxiety disorders, higher risk for suicide, and a diminished response to antidepressant treatments [4–7]. Understanding how ELA exerts its long-lasting effects on the brain will therefore be crucial to identify novel biological targets for early intervention or better treatment of psychopathologies that have their origin early in life.

In this review, we will discuss some of the neurobiological systems that are affected by ELA during sensitive periods of early postnatal development, and how changes in neural network maturation can have long-lasting effects on cognition and affective behavior. We will also discuss how genetic and environmental influences interact to confer individual differences in vulnerability and resilience to ELA, as well as potential novel approaches to treat or prevent the neurobiological and psychological sequelae of ELA. While factors influencing prenatal development are also crucial in shaping brain development and future (mental-) health outcomes [8–10] the focus of this review will be on ELA effects during early postnatal periods. For a comprehensive review on prenatal stress effects, see [11].

## ELA and psychopathology

### Evidence from clinical studies

The clinical consequences of ELA have been shown to depend on the timing and duration of the adversity, the type and number of ACEs, an individual’s genetic background, as well as social factors, such as lack of a supportive environment or marginalization [2]. While studies have found that ACEs have cumulative effects on risk for mental illness [1, 3], it is important to consider that simply adding the number of ACEs may mask potential differences between milder stressors (e.g., parental divorce) and stronger stressors (e.g., sexual abuse), and does not take their timing and duration into account. It is also still unclear whether different ACEs affect similar brain regions, behaviors, and pathologies. Investigating the consequences of specific ACEs is further complicated by the fact that they often co-occur. McLaughlin et al. [12] therefore proposed a dimensional system that categorizes different ACEs depending on their level of “threat” and level of “deprivation”. They argue that the dimension of “threat”, which is characterized by the experience of a negative stressor, is fundamentally different from the dimension of “deprivation”, which is characterized by a reduction in overall stimulation. According to this classification, ACEs such as abuse or natural disasters are thus high in the dimension of “threat”, while institutionalization and parental neglect are high on “deprivation”, and characterized by paucity of care, neglect, and malnutrition. Malnutrition often co-occurs with aberrant maternal care, and nutritional deficits have been shown to have similar consequences on cognitive functions as other early adversities. Deficits in early nutrition and metabolism may thus be important mediators of ELA effects [13, 14]. Some evidence suggests that “threat” and “deprivation” may have both differential and common effects on neural networks and cognitive processes. For example, children exposed to “threat” often have attention biases toward negative content and perceived threat, such as angry faces, whereas children exposed to “deprivation” instead have trouble distinguishing between emotions [15]. Children exposed to threat also have trouble discriminating between safe and threatening stimuli [16], and such increased fear generalization is associated with increased psychopathology in maltreated youth [17]. Similarly, children with a history of maternal deprivation are impaired in discriminating their own mother (“safe”) from a stranger (“threat”), as shown by indiscriminate friendliness and amygdala responses to either person [18]. This relationship between early life threat or deprivation and fear generalization may suggest a potential pathway from ELA to anxiety disorders or PTSD, in which fear overgeneralization is commonly observed [19]. Children exposed to either threat or deprivation also show problems with emotion regulation [20] and abnormal reward processing, shown, for example, by less approach motivation and lower neural response to reward [15, 21]. Since altered reward processing is a consistent finding in MDD [22], dysregulation in the reward circuitry may partially mediate the relationship between ELA and MDD [21].

Another dimension of ELA is the predictability or consistency of care and parental signals [23], which may be especially important for children raised by highly stressed or mentally ill parents. For example, being raised by a parent with MDD, confers 2–4 fold higher risk for psychopathology in offspring [24]. Having multiple generations of MDD further increases this risk to ~45% at age 9–10, and to even higher rates in adulthood [25, 26]. Such familial risk is likely mediated by both genetic and environmental mechanisms, which may include aberrant parenting practices, neglect, or abuse, but also inconsistent maternal signals, which are associated with impaired offspring cognitive development [27].

**Fig. 1:**
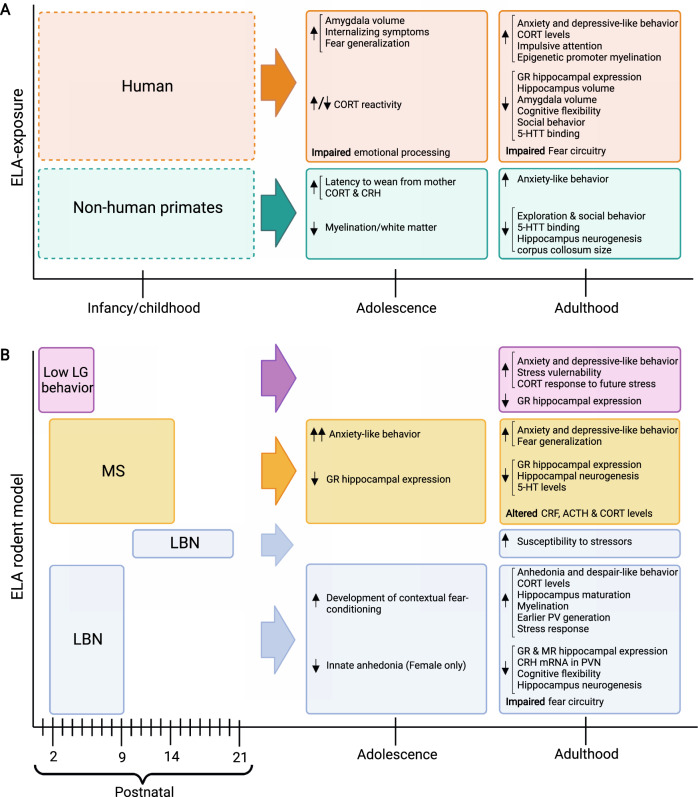
Behavioral, neurobiological, and physiological outcomes of postnatal ELA exposure in adolescence and adulthood. **A** ELA outcomes in humans and non-human primates. Dotted lines indicate the range of time ELA can occur. **B** ELA outcomes in rodents. The effects of different types of ELA models and the time period during which they are generally applied are indicated during P0–21. Adolescence and adulthood outcomes are listed for each species and model.

In summary, different types of adversities affect both different and shared biological systems and their associated cognitive processes and behaviors, as also summarized in Fig. 1A. Further research, including causality studies in animal models, is therefore needed to understand which neural networks are affected by specific ACEs, and how impairments in these neural networks may mediate ELA effects on psychopathology.

### Evidence from animal models

While human studies have revealed the impact of ACEs on psychopathology, they are limited by correlational inferences. Preclinical studies are invaluable in establishing causal relationships between adversity, brain function, and behavior, which may bear great potential to discover novel opportunities for therapeutic interventions aimed at targeting neurobiological mechanisms that are impaired by ELA. Some of these models will be discussed below, and are summarized in Fig. 1B.

#### Limited bedding and nesting (LBN)

In the LBN paradigm, rat or mice dams and their pups are placed on a wire mesh with 1/3rd of standard nesting material and 25% of bedding material for the first week of life (usually P2–9). These impoverished housing conditions prevent the dam from building a suitable nest that pups frequently fall out of. LBN conditions cause fragmented and unpredictable maternal care, as well as rough handling and stepping on pups, thereby potentially modeling “threat” and abusive behavior. In addition, while the overall amount of maternal care in the form of licking and grooming (LG) or total time spent with pups is unaffected, LBN causes reductions in pup body weight before weaning, suggesting potential effects on malnutrition that may result from “deprivation”, and that may mediate some of the effects of LBN on neurodevelopment [14, 28, 29]. Compared to offspring reared under control conditions, LBN exposed pups show altered somatic development and body growth, higher basal plasma corticosterone (CORT) levels, reduced corticotrophin releasing hormone (CRH) levels in the paraventricular nucleus (PVN) of the hypothalamus, reduced hippocampal expression of glucocorticoid receptors (GRs) and mineralocorticoid receptors, increased anhedonia and despair-like behavior, impairments in cognitive flexibility, and impaired long-term and short-term memory (Fig. 1B) [28–30]. Some of these consequences of LBN are more pronounced in females than in male offspring, and start to manifest in adulthood, but not yet in adolescence [29]. These findings indicate that adversity during the sensitive period from ~P2–9 has pronounced sex-dependent effects on cognitive and affective behaviors in mice that may be relevant for human psychopathology [31]. Interestingly, later exposure to LBN from P10–20, does not cause direct effects on behavior, but renders mice more susceptible to adult stressors, possibly through epigenetic and transcriptomic changes that are established during this period [32].

#### Maternal separation (MS)

In the MS model, dams are separated from their pups daily for 1–8 h over a 2–3 week period starting at P1–3 [33], depending on the experimental protocol. The repeated separation periods in this paradigm may be particularly relevant as a model of “deprivation”. A recent meta-analysis found that MS in rats increases offspring anxiety-like behaviors, which are more pronounced in adolescence than in adulthood (Fig. 1B) [34]. While MS appears to affect rats more strongly than mice in commonly used anxiety-like tasks [34], MS may affect mice specifically when assessing social behavior [35], or when combining MS and LBN exposure [36].

An important consideration with regards to MS is the duration of the daily separation bouts. The effects of MS on anxiety-like behaviors are more pronounced with longer separation periods and when individual pups are isolated from each other during separation from the dam [37]. In contrast, brief separation bouts of 15 min (referred to as “early handling”) have protective effects on offspring behavior [38], the neuroendocrine system [39], and hippocampus function [40]. These protective effects of handling are mediated by increased LG of pups by the dam following brief separation periods [39], emphasizing the importance of maternal care for offspring neurodevelopment and behavior.

#### Natural variations in maternal care

One important mediator for the effects of LBN, MS, and early handling is maternal behavior, which is fundamentally altered in these models [41]. Indeed, seminal work by the Meaney laboratory showed that natural variations in maternal care from P1–6 is critical for the long-term development of pups’ stress sensitivity and emotional development [39]. Specifically, pups raised by high LG mothers (high maternal care) show attenuated fear responses and improved learning [42], while pups raised by low LG mothers (low maternal care) show more anxiety-like behaviors, depressive-like behaviors, and increased stress vulnerability in adulthood (Fig. 1B) [39, 43, 44]. LG has been causally linked to offspring development by studies showing that cross-fostering pups of low LG dams with high LG dams reverses the behavioral and neuroendocrine impairments otherwise seen in pups raised by low LG mothers [45]. Moreover, daily tactile stimulation by stroking pups with a paintbrush from P1–5 [46] or from P3–21 [47] can mimic high LG and reverse behavioral impairments of low LG offspring, demonstrating that the quality of maternal care is a major influence on offspring development.

#### Non-human primate models

Mother-infant interactions in non-human primates show greater similarity to human relationships than rodent interactions. Some rhesus monkey mothers naturally exhibit abusive behavior toward their offspring, which involves throwing, crushing, or dragging offspring during the first 3 months of life [48]. In addition, the variable foraging demand (VFD) model has been used to create unpredictable rearing conditions by creating an experimental environment that requires the mother to use varying degrees of effort to obtain food for 12 weeks [49]. Both, abusive behavior and VFD, lead to higher offspring anxiety, excessive clinging to the mother, increased aggression, tantrums, lower exploratory behavior, and delayed social development (Fig. 1A) [50]. Moreover, infancy maltreatment and VFD have been shown to decrease myelination and white matter integrity in adolescence, increase CORT and CRH levels, and cause persistent cellular and molecular changes in the offspring hippocampus [51, 52].

## Sensitive periods of brain development

**Fig. 2:**
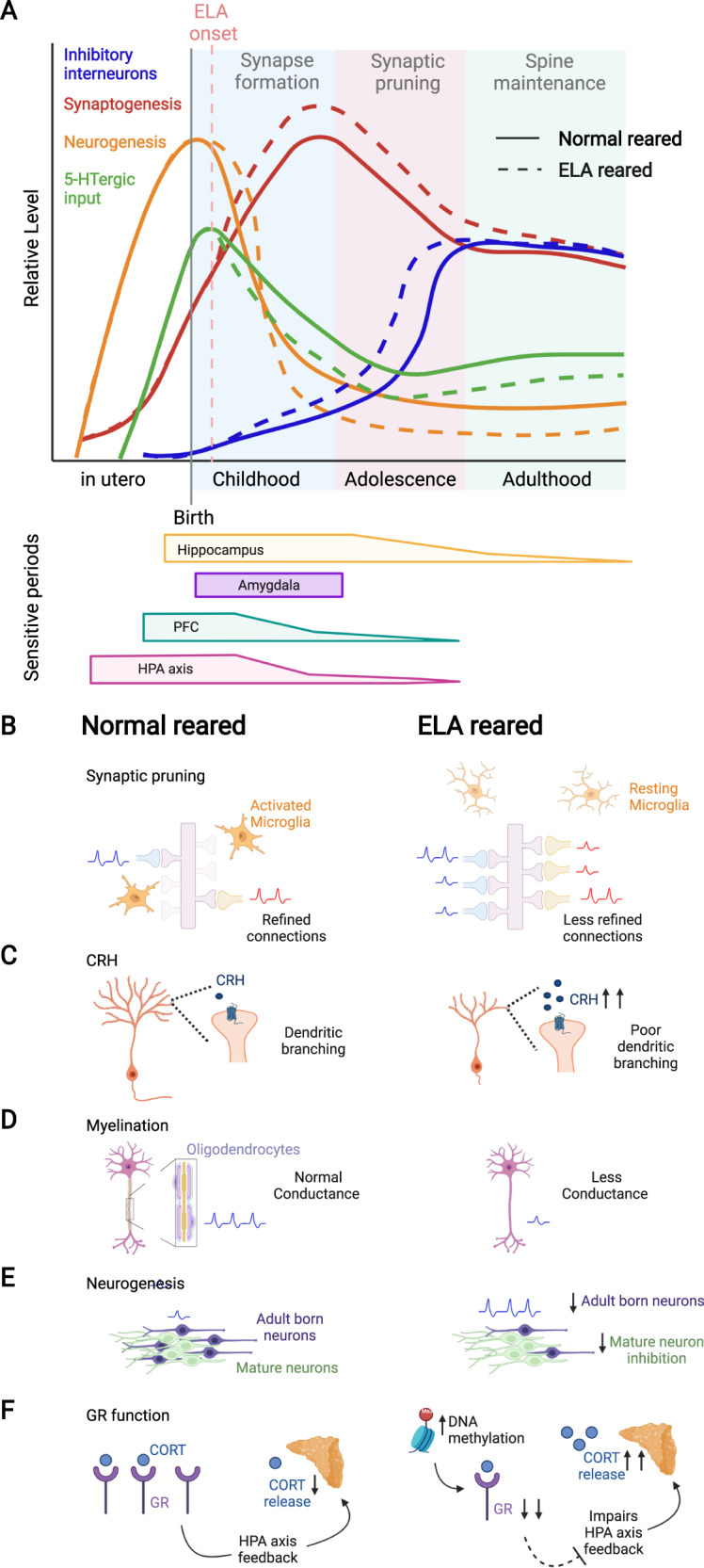
Plasticity of neural developmental processes throughout life. **A** Schematic showing the development of inhibitory interneurons (blue; adapted from [204]), synaptogenesis (red), neurogenesis (orange), and 5-HTergic input (green; adapted from [89]) from the prenatal period to adulthood. Solid lines indicate normal development, dotted lines indicate the effects of postnatal ELA. Childhood, adolescence, and adulthood correlate to peak time periods of synapse formation, synaptic pruning, and spine maintenance, respectively. The time windows for sensitive periods are indicated below for hippocampus (before age 13), amygdala (most pronounced volume changes during childhood), PFC (before age 2), and HPA axis (before age 2). The extended sensitive period for hippocampus development is determined by the continued neurogenesis in this region in adulthood (orange line). **B**–**F** Schematic depiction of ELA effects on neuroplasticity processes. **B** Synaptic pruning for normal reared individuals showing activated microglia engulfing weak synapses. ELA reduces microglia engulfment of synapses leading to less refined connections. Excitatory action potentials are indicated in blue, inhibitory action potentials are indicated in red. **C** ELA increases CRH levels resulting in poor dendritic branching. **D** ELA reduces myelination resulting in less conductance. **E** ELA reduces adult hippocampal neurogenesis, thereby potentially decreasing neurogenesis-mediated inhibition of the mature dentate gyrus granule neurons. **F** ELA decreases GR expression and impairs HPA axis feedback, subsequently increasing CORT release.

The brain is continuously altered by experience throughout life, but the formation and functional maturation of the brain is shaped particularly during the early postnatal period when circuit formation undergoes “sensitive periods”, during which circuits are particularly vulnerable to experience, or “critical periods”, during which experiences can lead to irreversible changes [53]. These periods of heightened plasticity are characterized by an interplay of several neurobiological processes, some of which we will describe below and in Fig. 2.

### Excitation and inhibition balance

The opening and closing of sensitive and critical periods are largely determined by the balance of inhibitory and excitatory neural input. Inhibitory neurotransmission continuously increases during early development [54] (Fig. 2A, blue line), and the enhanced plasticity of a sensitive/critical period ends once parvalbumin (PV) positive inhibitory interneurons mature and perineuronal nets (PNNs) accumulate [55]. One prominent example is the formation of the visual system, in which the beginning of the critical period for ocular dominance starts with an increase in inhibitory signaling [56], and can be shifted earlier by increasing GABAergic signaling through administration of benzodiazepines [57]. Similarly, removal of PNNs in adult rats, which resets PV+ neurons to a juvenile state, reinitiates a critical period in which ocular dominance can be induced [58], indicating that the timing of critical periods can be modified by changing excitation-inhibition balance. Indeed, ELA exposure reduces the number of PV+ interneurons and increases PNN accumulation around PV+ cells later in life, suggesting alterations in plasticity as a result of ELA [59, 60].

### Synaptogenesis and synaptic pruning

Synaptic development occurs primarily during sensitive periods in early life (~2–4 years in humans, ~P8–10 in mice) and in adolescence (~10–25 years in humans, ~P30–54 in mice; Fig. 2A, red line) [61]. An overproduction of synapses early in development initially creates a window for heightened plasticity that allows the brain to respond and adapt to the environment. Synaptic pruning then refines neural connections and shapes the development of complex circuits by strengthening highly active synapses and eliminating weaker, less active synapses (Fig. 2B) [61]. Microglia cells become more abundant during these periods and aid in the pruning process by phagocytosing weak synapses [62]. At the molecular level, microglia express innate immune system phagocytic receptors, such as complement receptor 3 (CR3/CD11b-CD18/Mac-1), whose ligand, C3, localizes to weak synapses and serves as a pruning signal for microglia [62]. Deficits in microglia CR3/C3 signaling result in more weak, immature synapses that produce weak synaptic transmission and reduce functional brain connectivity into adulthood [63]. This mechanism is particularly important for hippocampal synaptic maturation, which is significantly delayed when microglia function is disrupted by ELA exposure [63, 64]. Alongside microglia, astrocytes, and astrocyte-enriched engulfment receptor, MEGF10, help engulf and maintain synaptic connections and shape synapses in an activity-dependent manner [65]. In addition, CRH is required for refining dendritic arborizations through binding to CRH receptors that reside on dendritic spines and that are highly regulated by stress (Fig. 2C) [66]. Abnormalities in these cellular and molecular mechanisms by ELA can cause disrupted or excessive pruning, which has been linked to poor dendritic development, [67] neurological diseases, and early onset Schizophrenia [68].

### Myelination

In humans, myelination of white matter tracts begins in childhood and continues into early adulthood to facilitate axon conductance during neural circuit formation (Fig. 2D) [69]. White matter abnormalities in orphans and following social isolation during the first years of life impair hippocampal-prefrontal and fronto-striatal connectivity and are associated with impulsivity, and attention- and social deficits, and are not rescued with foster care replacement (Fig. 1A) [70]. Similarly, juvenile social isolation decreases corpus callosum size in rhesus monkeys, and mPFC myelination in mice. These effects are associated with impairments in working memory and social behavior, and can also not be rescued by social reintegration [71]. Interestingly, isolation stress in adulthood does not cause these same impairments, pointing to childhood as the sensitive period for adversity effects on myelination.

### Neurogenesis

Newly generated neurons undergo a period of heightened excitability within the first 4–6 weeks of their cellular development (Fig. 2E) [72]. These high levels of excitability enable new neurons to synapse onto other neurons more readily, and to out-compete older connections that have weaker synaptic input [73]. The time during which new, hyperactive young neurons are being formed thus likely contributes to the onset and duration of sensitive periods in different brain regions during prenatal and early postnatal development. Early life experiences may thus have greater influence on the long-term development of the brain than experiences in adulthood, as the rate of neurogenesis sharply declines after puberty. However, neurogenesis continues throughout adulthood in the dentate gyrus (DG) region of the hippocampus [74–76], suggesting that the sensitive period of development in the hippocampus is an extended process that may make this region more sensitive to experience-dependent influences in adulthood, as compared to other brain regions in which neurogenesis is restricted to prenatal and early postnatal life (Fig. 2A, orange line). Of note, some studies in humans have questioned the persistence of hippocampal neurogenesis past puberty [77], and more research in the field of human hippocampal neurogenesis in the adult brain is much needed [78, 79]. In addition to generating new neurons and refining synaptic connectivity, neurogenesis has been shown by us and others to contribute to input-dependent excitation/inhibition balance in the hippocampus, thereby likely determining its sensitivity to experiential influences, including stress [80–82]. ELA has been shown to decrease hippocampal neurogenesis in adult rodents [83, 84], thereby potentially contributing to heightened stress vulnerability later in life (see also “ELA effects on the hippocampus”).

### Serotonin (5-HT) effects during development

The 5-HT system mainly develops prenatally, starting at 5 weeks of gestation in humans (~E12 in rodents) when proliferating 5-HT precursor cells first emerge [85]. Serotonergic projections start to extend to cortical regions at 8 weeks gestation in humans, and 5-HT levels peak at 5 years before declining to adult levels (Fig. 2A, green line) [86]. Serotonin transporter (5-HTT) expression continues to increase until age 18 and then decreases at a rate of ~1% per year [87], possibly reflecting changes in axonal branching of 5-HT neurons [88]. In mice, serotonergic projections mature around P2–11 when cortical 5-HT levels peak, but the patterns of innervation continue to develop until P21 [89]. The development of the 5-HT system is in part mediated by 5-HT binding to 5-HT_1A/1B_ autoreceptors, which refine the number of 5-HT neurons in the raphe nuclei [90], as well as binding to 5-HT_1B/1D_ heteroreceptors, which promote axon guidance [91].

Some of the functional roles of 5-HT during development include regulation of neurogenesis, synaptogenesis, neural connectivity, myelination, and synaptic remodeling (for a comprehensive review, see [87]). While a multitude of studies has shown anxiolytic effects of 5-HT [92], some have shown anxiogenic effects during early development [93]. In mice, increasing 5-HT signaling during P2–11, but not after P12, enhances anxiety- and depressive-like behavior in adulthood [93], and similar effects are observed in humans at analogous developmental stages [94], indicating that 5-HT can have anxiogenic effects during early phases of development. ELA has been shown to affect several components of the 5-HT system, which will be discussed in detail in section “ELA effects on the 5-HT system”.

### Hypothalamus-pituitary-adrenal (HPA) axis responsiveness during development

While the HPA axis responds robustly with an increase in CORT levels to stressful stimuli in adulthood, its responsivity is attenuated early in life during a “stress-hyporesponsive period (SHRP)” in both humans (until ~age 2) and rodents (until ~P14) [95, 96]. This SHRP might serve a neuroprotective purpose by shielding the brain from the detrimental effects of excessive CORT on cell survival, neurogenesis, and synapse formation during sensitive periods of early development [97, 98]. The SHRP is characterized by a hypoactive adrenal cortex that only secretes low levels of CORT, and by a pituitary gland that is highly sensitive to GR-mediated negative feedback inhibition of the HPA axis (Fig. 2F). Moreover, CRH levels in the PVN and GR levels in hippocampal CA1 are high during the SHRP and peak at P12 in rodents [95]. In addition to these physiological characteristics, the nurturing environment provided by the mother may contribute to a buffering of offspring stress responses during the SHRP. Disturbances to the quality and quantity of maternal care during the SHRP may thus render offspring especially vulnerable to ELA-induced neuronal changes. While the SHRP may have evolved specifically to protect the developing brain from noxious influences during sensitive developmental periods, ELA exposure can “break” the SHRP, increasing CORT levels, and leading to persistent changes in HPA axis development and function [95, 99].

## ELA effects on neural circuitry during sensitive periods

**Fig. 3:**
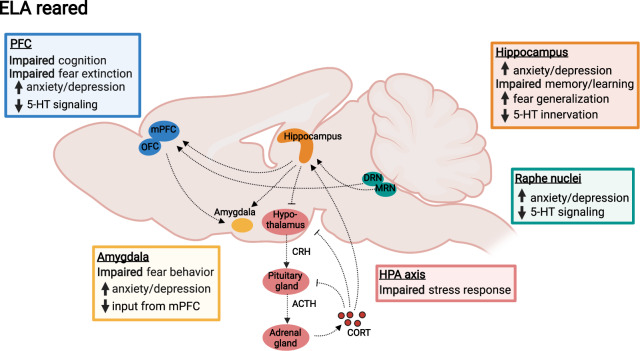
ELA effects on neural circuitry. Shown are postnatal ELA effects on brain regions and neural circuits commonly implicated in psychopathology, such as the hippocampus, mPFC, OFC, amygdala, the HPA axis, and serotonergic signaling from the raphe nuclei (DRN and MRN) to cortical and limbic regions. Colored boxes denote ELA effects on specific functions of each brain region related to cognition and affective behavior.

The pronounced effects of ELA on brain function and behavior likely result from changes in several of the above-described processes underlying neural circuit formation during sensitive periods. Below, we will discuss some of the effects of ELA on brain regions and neural circuits that have been implicated in psychopathology (Fig. 3).

### ELA effects on the hippocampus

The hippocampus is involved in a range of psychopathologies, and hippocampal volume and microstructure are decreased in individuals with, or at high risk for, MDD, PTSD, anxiety, and schizophrenia [100, 101]. ELA leads to smaller hippocampal volume especially when experienced before age 13 (Fig. 1A) [102], suggesting that hippocampus-dependent memory and emotion regulation may be particularly sensitive during this period (Fig. 3). Human postmortem studies have shown that the DG is smaller and granule neurons are fewer in MDD patients who experienced ELA, while larger DG volume is associated with resilience in individuals who experienced ELA but did not develop MDD [103]. Interestingly, individuals at high familial risk for depression have lower DG volume and microstructure, which predicts future, but not past or current, depressive symptoms [100, 104]. Together, these findings suggest that decreased DG structure is not only a consequence of psychopathology but indeed an ELA-induced risk factor for disease.

Rodent ELA models support these findings, and have shown reduced hippocampal volume and neurogenesis, disrupted dendritic structure and connectivity, reduced spine density, increased maturation of mossy fiber synapses, and impaired long-term potentiation [67, 105, 106]. Some of these effects may be mediated by elevated CORT levels that impair structure and function of hippocampal neurons following ELA [29, 98]. Interestingly, LBN conditions increase neural maturation of the early postnatal offspring hippocampus, similar to findings in humans. This precocious maturation includes an earlier rise in myelination, in the ratio of NMDA receptor subunit NR2a over NR2b, and in PV+ neurons in the DG [29, 107]. These data thus suggest that ELA may shift the opening and closing of sensitive periods by accelerating neural development in the hippocampus.

In addition, ELA has been shown to decrease hippocampal neurogenesis in adult rats after MS [108], in mice following LBN despite an initial spike in neurogenesis at P9 [84], and in non-human primates following VFD [109]. ELA-exposed female mice show a reduction in neurogenesis during puberty that normalizes by adulthood, while neurogenesis in ELA-exposed males increases slightly until puberty onset and then drops in adulthood. These findings suggest that ELA effects on neurogenesis are sex dependent [110]. Adult hippocampal neurogenesis and DG function are important for contextual fear discrimination [111], behavioral and neuroendocrine stress responses [80, 112], stress-induced anxiety and depressive-like behaviors [80, 113], and for some, but not all, behavioral effects of antidepressants [114, 115]. Impairments in neurogenesis may thus be a potential mechanism by which ELA exerts long-lasting effects on stress responses and antidepressant responsiveness.

### ELA effects on the HPA axis

In some depressed patients, CORT levels are chronically elevated, and diurnal CORT rhythms, CORT responses to stress, and CORT awakening responses are dysregulated [114]. These disturbances in HPA axis functionality may have their origin early in life, as increased CORT responses following childhood maltreatment moderate some of the effects of maltreatment on depressive symptomatology (Fig. 3) [116, 117]. Variations in parental care are also associated with individual differences in neuroendocrine responses and emotional reactivity [118]. These ELA effects on the HPA axis may be mediated in part by the GR, which shows decreased expression [119] and increased promoter methylation in peripheral blood samples [120] and in postmortem hippocampus tissue of subjects with a history of severe ELA (Figs. 1A and 2F) [121]. It is important to note that both blunted and elevated CORT responses are associated with mental illness, and both have also been reported as a result of ELA, possibly depending on the timing of the adversity or co-occurrence of (psycho-)pathologies. For example, blunted CORT responses to injections have been observed in 12-month old infants of mothers with unpredictable maternal signals [122]. A potential sensitive period of HPA axis development around age 2 is suggested by studies showing that orphans who remain institutionalized beyond age 2 show blunted cortisol response to the Trier Social Stress Test, which are not observed in children who are placed in foster care before age 2 [123].

Rodent studies support these clinical findings and have reported impairments in HPA axis function following MS, including altered CRH, ACTH, and CORT levels, as well as decreases in hippocampal GR expression that last into adulthood (Figs. 1B and 2F) [124–126]. In LBN-exposed rodents, GR expression and CRH levels are decreased in the PVN, leading to impaired HPA axis negative feedback [28, 127]. Moreover, PVN CRH neurons are more excitable after LBN [128] and may thus promote prolonged responses to stress in adulthood [129]. On the contrary, increased and more predictable maternal care decreases glutamatergic input onto CRH neurons, thereby reducing stress responses later in life [130]. Furthermore, low maternal LG during the first week of life reduces offspring hippocampal GR expression, impairs HPA axis negative feedback, and enhances CORT responses to stress that are rescued by cross-fostering or manual brushing [43, 44, 131, 132].

Based on these converging clinical and preclinical findings, ELA has complex effects on HPA axis function. This HPA axis dysregulation in turn causes long-lasting impairments in neuroendocrine stress responses that potentially contribute to various aspects of neural circuit malfunction that contribute to future psychopathology.

### ELA effects on fear circuits

Abnormal fear responses are observed in children and adults with a history of ELA, and these impairments may be caused by a dysregulation of neural responses to fearful stimuli in the amygdala (Fig. 3) [133, 134]. At a structural level, amygdala volume is increased in children following ELA, but decreased in adulthood (Fig. 1A) [135]. Similar to the aforementioned effects of ELA on hippocampus development, this effect on amygdala volume has been hypothesized to reflect precocious maturation [136], which may ultimately result in accelerated volume loss [137]. In contrast, adversity in adolescence decreases amygdala volume, pointing toward differences in fear circuit regulation depending on the timing of adversity [138]. Impaired emotion regulation in adults with a history of ELA has been suggested to result from altered connectivity between the amygdala and the mPFC [139]. However, in institutionalized children with low separation anxiety amygdala-mPFC connectivity develops earlier than in institutionalized children with high anxiety or controls, suggesting that ELA may in fact enhance or accelerate emotion regulation in resilient individuals [134].

In rodents, MS and LBN increase fear expression and fear generalization, which both depend on hippocampus and amygdala circuits (Fig. 1B) [140, 141]. These findings suggest that ELA in rodents causes fear overgeneralization similar to observations in humans. This ELA effect on fear overgeneralization is at least in part mediated by the GR, suggesting a contribution of the HPA axis to fear circuit regulation [14, 142]. ELA also accelerates amygdala development and fear circuit maturation in rodents, and LBN exposed mice develop the ability to suppress freezing responses to conditioned fear 1 week earlier than controls (at P22 instead of P28) [29, 143]. In addition, auditory fear conditioning is impaired following LBN and can be rescued by optogenetic inactivation of PV neurons in the BLA [144], suggesting that ELA effects on inhibitory circuits in the BLA may underlie impairments in fear learning.

### ELA effects on neural circuits of cognition

The regulation of cognitive function by connectivity between the hippocampus and cortical structures is formed during childhood and adolescence in humans, and during the first few weeks of life in rodents [145]. Stress during these periods can permanently alter the development of neural connections between the hippocampus, mPFC, and orbitofrontal cortex (OFC), which are crucial for cognitive function (Fig. 3) [106, 146]. Indeed, children exposed to maltreatment or maternal depression show deficits in executive functions, such as cognitive flexibility, inhibitory control, working memory, and long-term memory (Fig. 1A) [147]. Increased CORT awakening responses resulting from ELA are also associated with decreased problem solving and planning [148]. Accordingly, similar to the potential sensitive period of HPA axis development, cognitive function is often impaired in institutionalized children unless they entered the foster care system before age 2 [149].

Recent epidemiological studies show that ELA exposure increases cognitive aging, leading to cognitive decline and elevated risk for developing Alzheimer’s disease [150, 151]. This effect of ELA on cognitive aging may be mediated by impaired HPA axis function, deficits in nutrition, altered inflammatory responses, impaired dendritic and synaptic formation, deficits in neural plasticity, and changes in proteins such as early growth response protein 1, activity regulated cytoskeleton-associated protein (Arc), and repressor element-1 silencing transcription factor [151]. One specific example includes the Dutch famine study showing increased cognitive decline following malnutrition exposure during early development [152]. Thus, age-related brain disorders, such as cognitive impairments and Alzheimer’s disease may have their origin already early in life.

Rodent studies have shown that impairments in hippocampus and PFC structure and function resulting from ELA cause long-term deficits in memory tasks, such as contextual fear conditioning, the Morris water maze, novel object recognition, and object location learning tasks [105, 125, 140]. Furthermore, female mice exposed to LBN have deficits in reversal learning, a crucial form of cognitive flexibility, which is caused by a decrease in the numbers and function of PV interneurons in the OFC and mPFC [144].

### ELA effects on the 5-HT system

Serotonin signaling is impaired in a number of mental disorders and a main target of psychiatric medications [92]. At the mechanistic level, rodent studies have indeed shown that 5-HT deficiency in the brain increases vulnerability to stress in adulthood [153]. While the 5-HT system primarily develops prenatally, early postnatal stress may impact serotonergic innervation and circuit development by affecting the maturation of 5-HT projections during the early postnatal period (Fig. 3). This is supported by positron emission tomography studies, which have shown that childhood abuse in humans, and MS in rhesus macaques, are associated with lower 5-HTT binding across brain regions, including the hippocampus and amygdala, possibly reflecting lower axon density of serotonin neurons [154, 155]. While some studies have found that ELA reduces CSF concentrations of the 5-HT metabolite, 5-hydroxyindoleacetic acid (5-HIAA) [156, 157], others have reported increased 5-HIAA levels that inversely correlated with hippocampal volume, possibly due to increased raphe 5-HT levels that may in turn inhibit serotonergic neurons through excessive 5-HT_1A_ autoreceptor activation [158].

In rodents, 3–6 h of daily MS decreases 5-HT levels in the hippocampus and hypothalamus of adult rats (Fig. 1B) [159]. On the contrary, stronger stress in the form of two bouts of MS for 3 h each day increases 5-HT levels in the hippocampus, amygdala, and PFC in some studies [160]. Importantly, this increase in 5-HT is accompanied by a decrease in 5-HT metabolic turnover, as indicated by lower 5-HIAA/5-HT ratios [160]. This finding potentially indicates that MS may reduce synaptic release of 5-HT, resulting in decreased metabolic turnover and an accumulation of 5-HT in intracellular synaptic vesicles of serotonergic dendrites in limbic or cortical projection areas. Studies have also shown that ɑ1 and CRF2 receptor mediated excitation, as well as 5-HT_1A_ and CRF1 receptor mediated inhibition of serotonergic neurons are impaired following MS [161]. This finding could potentially be of value for therapeutic intervention, considering that CRF1 receptor inhibition prevents ELA effects on PFC dendritic development and cognitive impairments [162]. In addition, MS followed by post-weaning social isolation decreases 5-HT terminal density in the rodent hippocampus [163], and foot shocks can reduce the number of serotonergic cell bodies early in life [164].

The downstream effects of 5-HT in limbic and cortical projections areas are mediated by 5-HT receptors. Accordingly, 5-HT_1A_ heteroreceptors in the hippocampus and mPFC are reduced by MS at P7 [160]. Considering that 5-HT_1A_ heteroreceptors in these regions during P5–21 are crucial for the development of normal anxiety levels in adulthood [165], downregulation of 5-HT_1A_ by ELA during this period may impact the proper development of circuits underlying the regulation of anxiety-like behavior in adulthood [166].

Together, these results suggest an overall decrease in the function of the 5-HT system as a result of ELA (Fig. 2A, green lines).

## Individual differences in susceptibility and resilience to ELA

It is important to note that not every individual who is exposed to ELA will ultimately develop psychopathology. While our understanding of individual differences in vulnerability to ELA is still in its infancy, resilience determining factors include both genetics and environmental factors, as well as gene-by-environment (GxE) interactions.

One of the perhaps best-known examples of such a GxE interaction is that individuals with a history of childhood adversity that carry the short (S) allele of the 5-HTT gene are more likely to develop a depressive episode than individuals who experienced childhood adversity and carry the long (L) allele of the gene [167]. S allele carriers also show reduced CSF 5-HIAA levels [168] and higher amygdala activity than L allele carriers [169]. Similarly, rhesus macaques with the S allele who experience ELA show heightened stress responses, reduced serotonergic function, and greater anxiety than L allele carriers [170], and mice with disrupted 5-HTT show enhanced ACTH levels in response to stress [171]. Rodent studies have also shown that knockdown of the 5-HT_1A_ autoreceptor on serotonergic neurons rescues juvenile stress effects on avoidance behavior [172]. This finding is in line with studies showing that stress susceptibility is increased in mice with high levels of 5HT_1A_ autoreceptors [173], and studies showing that brain 5-HT deficiency causes increased susceptibility to adult- and early life stress [174].

Other candidate genes implicated in vulnerability to ELA include key regulators of the HPA axis. For example, GxE interactions have been found for the GR polymorphisms 22/23EK and 9beta and childhood adversity, which result in increased risk for depression [175]. Moreover, interactions between early trauma and the FKBP5 polymorphism, rs1360780, predict lifetime PTSD in the absence of a main genetic effect of FKBP5 genotype [176].

It is important to note that controversy surrounding the reproducibility of candidate gene studies has emphasized the need for more genome-wide association studies (GWAS) in psychiatry [177]. While there is currently no GWAS on susceptibility to ELA, some studies have evaluated the interaction between GWAS-derived personal polygenic risk scores (PRS) for psychopathology and ELA, or assessed genetic overlap between personality traits and resilience. For example, childhood trauma and personal cumulative genetic risk for depression, as measured by PRS, both predict depression individually and interact to increase risk [178], and polygenic risk for neuroticism interacts with ELA to predict MDD in adulthood [179].

Unlocking the neurobiological factors that confer resilience to ELA may be key to developing novel therapies to alleviate the burden of ELA on mental illness. Interestingly, a GWAS of resilience in combat veterans found significant risk loci associated with the doublecortin family, which is implicated in hippocampal neurogenesis [180]. Considering that hippocampal neurogenesis is one important mechanism of stress resilience [80, 112], and that ELA reduces neurogenesis [108], increasing neurogenesis may provide new avenues to rescue ELA effects on psychopathology. While in humans the persistence of adult hippocampal neurogenesis is still debated [74, 77], interventions before puberty, when some neurogenesis is consistently detected in human postmortem tissue [75, 77, 78, 181], could be particularly useful to prevent ELA effects. Additional resilience-promoting factors may include cellular and molecular regulators of the 5-HT system or the HPA axis, such as 5-HT receptors and GRs, nutritional interventions, high social support, physical activity, or cognitive flexibility and emotion regulation training [182]. Improving social support systems, diet, activity levels, and neural circuits underlying cognitive flexibility, emotion regulation, and stress response regulation may thus be promising new avenues to improve resilience in vulnerable populations.

In addition to the prevailing view that ELA increases vulnerability to stress that is experienced later in life, the match-mismatch hypothesis of stress responsivity suggests that ELA exposure can also promote resilience to future stressors by “preparing” an individual to cope better with a life in a highly adverse environment. This hypothesis is supported by studies showing the complex interaction between sex, timing, and type of the stressor in early life and adulthood of the exposed individual, all playing a role in shaping future stress responses and their underlying neurobiological substrates [140, 183].

## Epigenetic mediators of ELA on neural circuit function

Epigenetic mechanisms have repeatedly been implicated in mediating long-lasting effects of early experiences, including in the form of DNA methylation or histone modifications that can alter gene expression (for a comprehensive review, see [184]).

Genome-wide and long-lasting epigenetic changes have been shown to be caused by ELA in the human hippocampus [185]. One prominent example is increased GR methylation and reduced GR expression in postmortem hippocampus tissue of individuals with a history of maltreatment [121]. This change in GR expression has been linked to early life epigenetic reprogramming of the HPA axis, which has been demonstrated by rat studies in which low LG causes hypermethylation of the hippocampal transcription factor nerve-growth factor-inducible factor-A (NGFI-A) consensus sequence at the GR exon 1_7_ promoter during the first week of life. This effect is stable until at least P90 and can be reversed by cross-fostering to a high LG dam within 12 h of birth [43]. Offspring raised by low LG dams also show less acetylation at the histone H3K9 residue. These epigenetic changes reduce the accessibility of the GR promoter, causing a threefold lower binding of NGFI-A that results in reduced hippocampal GR expression, impaired HPA axis negative feedback, and greater CORT responses to stress. This epigenetic programming of the GR1_7_ promoter is mediated directly by NGFI-A [186], which is activated by 5-HT and thyroid hormones that are released in response to the tactile stimulation of LG [187]. Similarly, MS causes GR hypermethylation in the hippocampus [188] and in hypothalamic CRH-producing neurons, where it causes blunted CRH upregulation during stress [189]. In addition, MS causes hypomethylation of the *Avp* gene in the PVN [190], and of the *Pomc* gene in the pituitary [191], which increase AVP and ACTH expression, respectively, resulting in sustained HPA axis hyperactivity. ELA effects on nutrition and metabolism may also play an important role in epigenetic regulation of hippocampal function, cognition, and mental illness, as foods high in methionine, folate, betaine, and choline contain dietary methyl donors that influence the epigenetic machinery [13]. Increased nutritional interventions early in life can rescue ELA effects, suggesting that dietary micro- and macro nutrients may be promising strategies to explore for the prevention and/or treatment of early stress effects on the brain [192, 193]. Some of these epigenetic changes, however, seem to be dependent on which combination of strain, type of stress, and sex is being examined. Importantly, exposure of developing human hippocampal neurons to high levels of CORT causes long-lasting changes in global DNA methylation in vitro and reduces cell proliferation and neural development [97, 98, 194]. Epigenetic regulatory mechanisms have also been described for functional glucocorticoid response elements in *fkbp5*, which are demethylated in peripheral blood mononuclear cells (PBMCs) of individuals with a history of childhood trauma and in CORT-exposed developing human hippocampal neurons [176].

ELA also changes expression of receptors implicated in neural excitation and inhibition, such as GAD1 and mGluR1. These expression changes may be epigenetically mediated, as pups raised by low LG dams have increased DNA methylation of the GAD1 gene and the mGluR1 gene, as well as decreased acetylation of H3K9 at the mGluR1 promoter [195, 196].

Evidence for epigenetic regulation of the 5-HT system can be derived from studies showing that the 5-HTT gene is hypermethylated in non-human primates following ELA, resulting in stress hyperreactivity and long-lasting impairments in physical health [197]. Moreover, MS in mice increases acetylation of histones at loci associated with G-proteins, which mediate 5-HT receptor signaling [198], suggesting that downstream molecular pathways of 5-HT signaling may be epigenetically regulated by ELA [184].

ELA from birth through age 7 is associated with higher internalizing problems that are mediated by an inflammation-related epigenetic PRS, which was derived from data from an epigenome-wide association study [199]. A role for epigenetic regulation of the inflammatory system has been demonstrated by studies showing that childhood adversity causes DNA methylation changes in immune system genes, such as hypomethylation of the IL6 promoter in PBMCs [200]. In rodents, ELA from P14–21 decreases global DNA methylation in microglia and increases microglia activation [201]. These epigenetic changes, together with increased pro-inflammatory cytokine release, may result in long-lasting microglia dysfunction and subsequent impairments in synaptic pruning and myelination during sensitive developmental periods. [202] Indeed, inhibiting microglia activation in mice has been shown to rescue ELA effects on behavior [203].

Considering the wide-ranging and long-lasting effects of ELA on the molecular regulation of the neuroendocrine, serotonin, and inflammatory system, a better understanding of the epigenetic regulation of these systems may have great potential to reveal new molecular targets for advanced treatments or preventative strategies.

## Conclusion

ELA has pervasive effects on the development and function of neural circuits, which increases the risk for psychopathology across the lifespan. Adversity that is experienced during sensitive periods of early postnatal development can disrupt cellular and molecular processes that regulate the normal formation of neural networks underlying cognition and affective behavior. These effects of ELA depend on the timing, the modality, and the number of adverse experiences, but also on genetic risk factors, sex, nutrition, and the social support of the individual, which can shape vulnerability to developing psychopathology. Understanding how ELA disrupts the complex interplay between the neuroendocrine system, the serotonin system, and neural circuits of emotion regulation, fear, and cognition, is a major challenge for modern neuroscience and psychiatry that will have to take into account the molecular regulation of these systems as well as the developmental trajectory of individual brain regions. Integrating clinical and preclinical research findings will be crucial to understanding which neurobiological mechanisms causally mediate ELA effects on psychopathology in animal studies that appropriately model crucial aspects of the human condition.
